# Supplementary material for: Improved protein structure refinement guided by deep learning based accuracy estimation
Source: Nat Commun. 2021 Feb 26;12:1340. doi: 10.1038/s41467-021-21511-x (PMC7910447; doi:10.1038/s41467-021-21511-x)
Supplement: Supplementary file 1 — Supplementary Information [file 41467_2021_21511_MOESM1_ESM.pdf]

Supplementary Information

Improved protein structure refinement guided by deep learning based accuracy estimation

Naozumi Hiranuma[1,2], Hahnbeom Park[1], Minkyung Baek[1], Ivan Anishchanka[1], Justas Dauparas[1], and David Baker[1,3,*].

1 - Department of Biochemistry and Institute for Protein Design, University of Washington, WA, USA

2 - Paul G. Allen School of Computer Science & Engineering, University of Washington, WA, USA

3 - Howard Hughes Medical Institute, University of Washington, WA, USA

These authors contributed equally: Naozumi Hiranuma, Hahnbeom Park

Correspondence to: *dabaker@uw.edu

Supplementary Figures

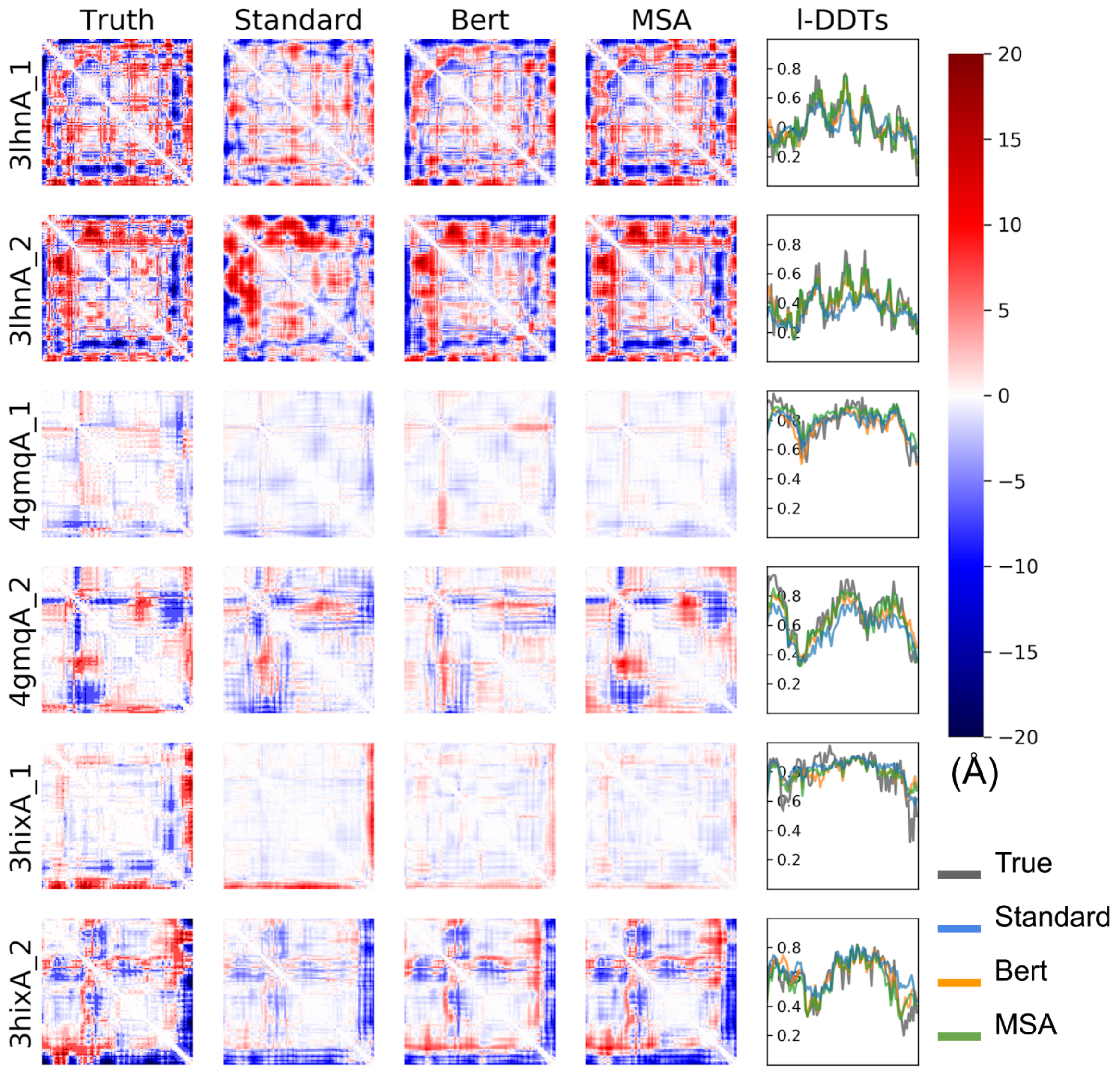

Supplementary Figure 1: Example estograms and C_β l-DDT score prediction from DeepAccNet Standard, Bert and MSA. Model predictions for the same set of decoys from Figure 2 (3lhnA, 4gmqA, 3hixA; size 108, 92 and 94 respectively). The first column shows true maps of errors, the second to fourth columns show predicted maps of errors, and the last column shows predicted and true C_β l-DDT scores. The i, j element of the error map is the expectation of actual or predicted estograms between residues i and j in the model and native structure. Red and blue indicate that the pair of residues are too far apart and too close, respectively. The color density shows the magnitude of expected errors.

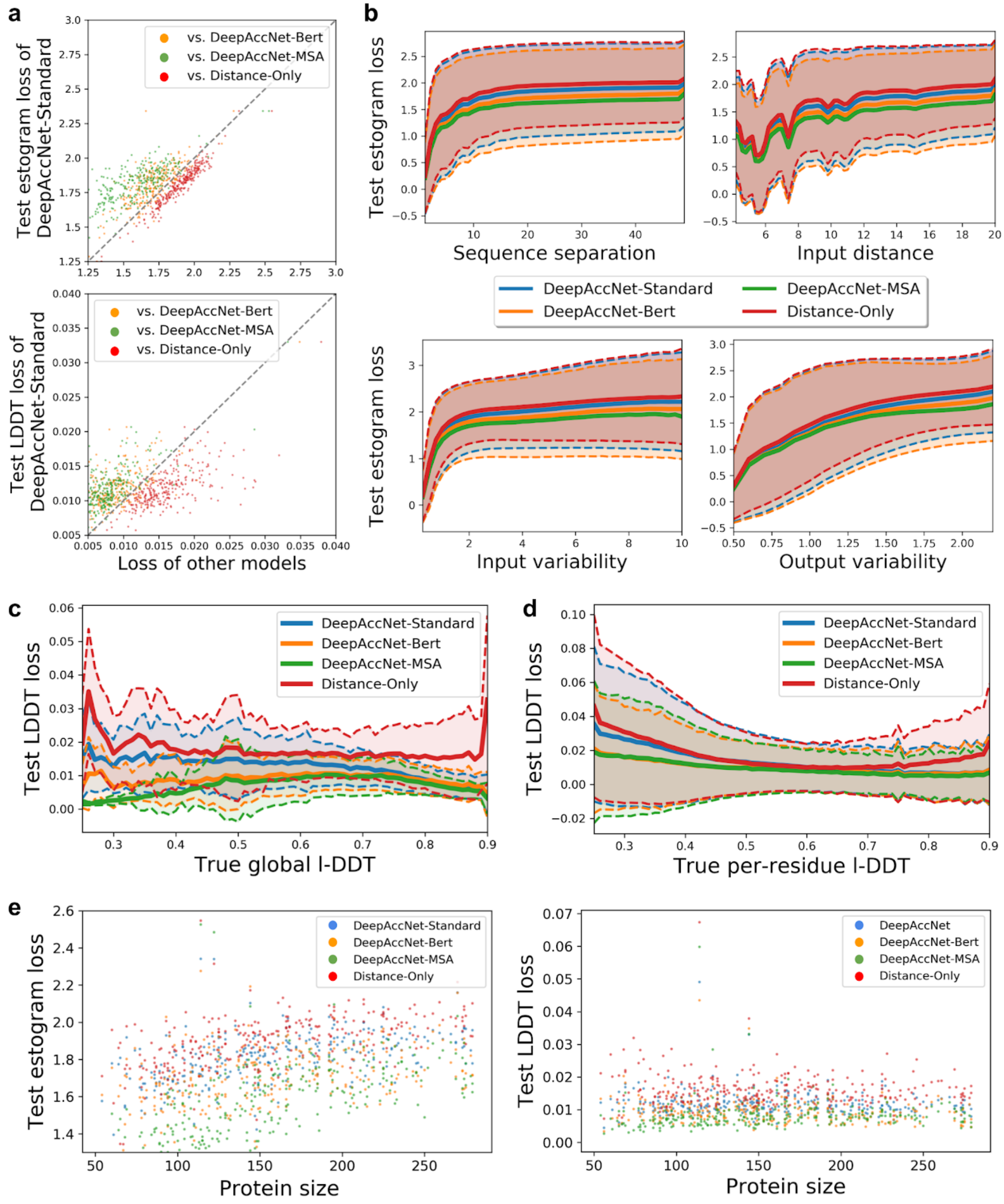

Supplementary Figure 2. a) Comparison of the variants of DeepAccNet and distance-only network on predicted estograms (top) and C_β l-DDT scores (bottom). Each dot represents the loss for a single protein averaged over all decoys. Lower loss values indicate better performance. Estograms are evaluated by cross-entropy loss, and per residue C_β l-DDT scores are evaluated by mean-squared error. **b**) Test estogram loss plotted against four conditions; sequence separation, input distance, input variability (standard deviation of input distance across decoys from the same target), and output variability (entropy of true estogram across decoys from the same target). The loss values are binned in terms of x-axis properties. The mean value at each bin is shown on the y-axis, and the range of one z-score is shown with the shaded area. **cd**) Dependence of C_β l-DDT score loss on true C_β l-DDT per-model (**c**) and

per residue (**d**). Loss values are binned in terms of the true C_β l-DDT scores. The mean of loss values at each bin is shown on the y-axis as a solid line, and the range of one Z-score is shown with the shaded area. **e**) Dependence of estogram (left) and C_β l-DDT score per residue (right) loss on protein size. Each dot is an average loss value for a single target protein over all decoys.

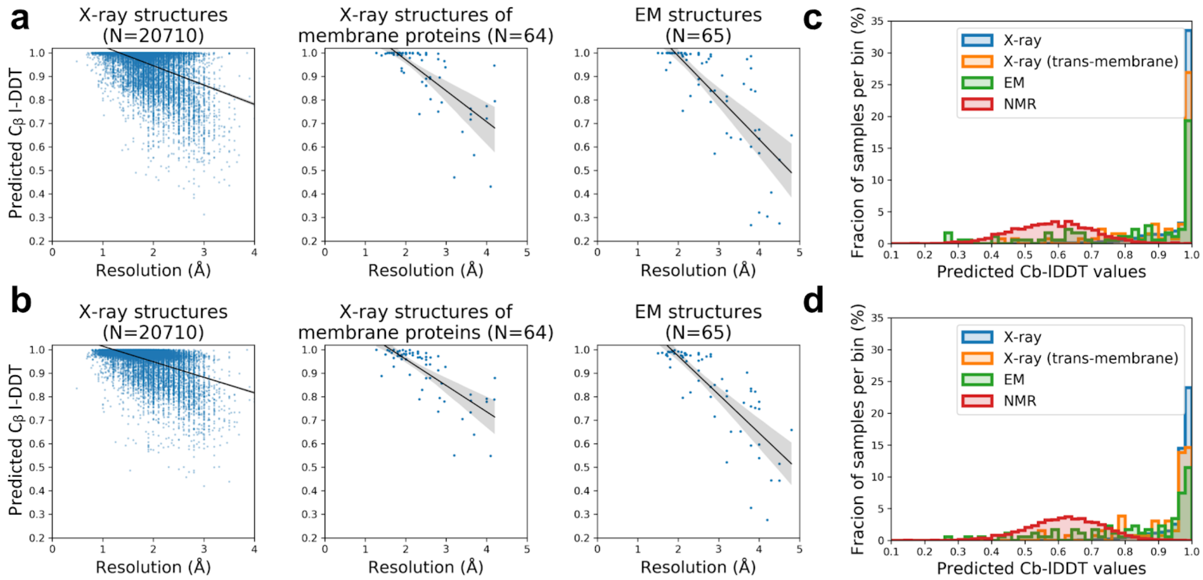

Supplementary Figure 3. ab) Predicted C_β l-DDT by DeepAccNet-Bert (**a**) and DeepAccNet-MSA (**b**) correlates with resolutions for X-ray structures (left; Spearman-r 0.43 and 0.44 with p-value < 0.0001 for the Bert and MSA variants, respectively), X-ray structures of transmembrane proteins (middle; Spearman-r 0.73 and 0.74 with p-value < 0.0001 for the Bert and MSA variants, respectively), and cryoEM structures (right; Spearman-r 0.82 and 0.84 with p-value < 0.0001 for the Bert and MSA variants, respectively). **cd**) X-ray structures have higher predicted l-DDT values by DeepAccNet-Bert and -MSA than NMR structures.

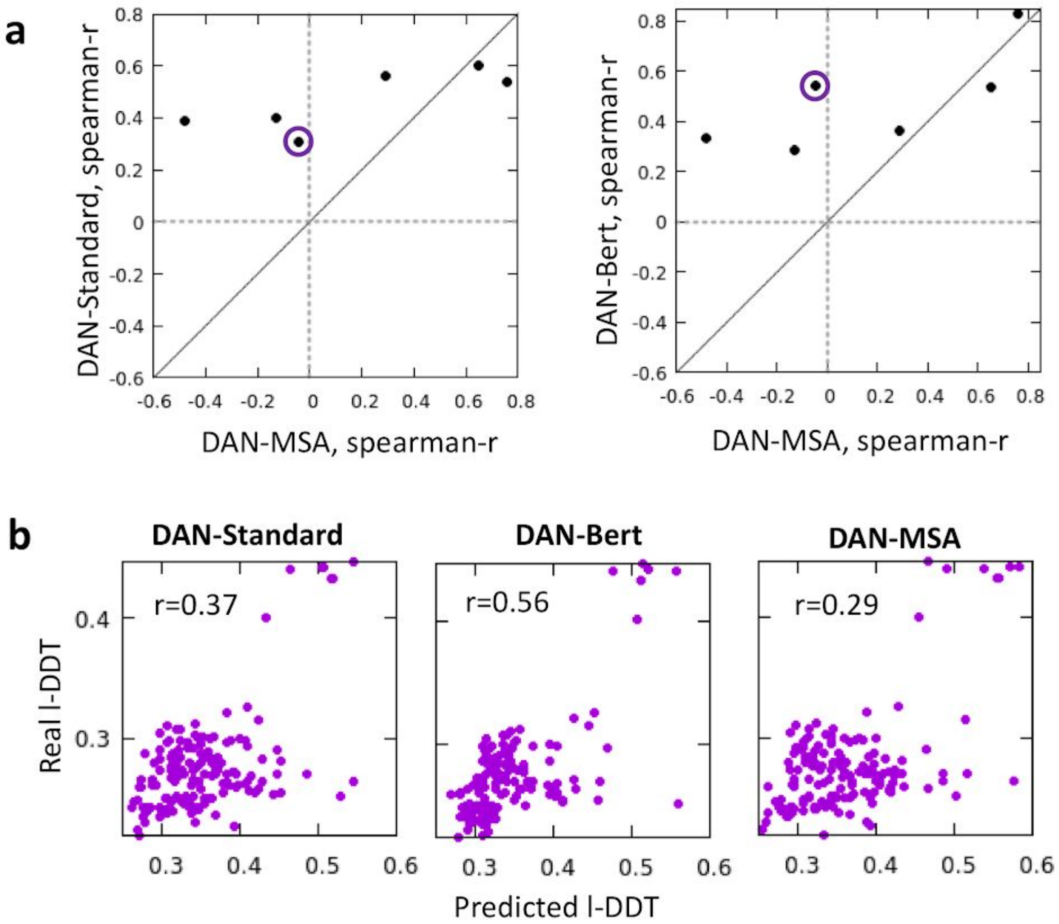

Supplementary Figure 4. DAN-Bert and DAN-Standard outperform DAN-MSA when protein has no homologous sequence information. a) Global EMA results of 6 targets from CASP14 which had no homologous sequence (UniClust30 [1] January 2020). For each target, Spearman-r between the predicted and the actual C_β l-DDT across 150 models generated by CASP14 participants is shown. left) DAN-MSA versus DAN-Standard, right) DAN-MSA versus DAN-Bert; DAN-MSA on the x-axis and the other on the y-axis. **b**) Scatter plots of EMA results by DAN-variants on a CASP14 EMA target T1043 (highlighted by purple circles in the panel (a).

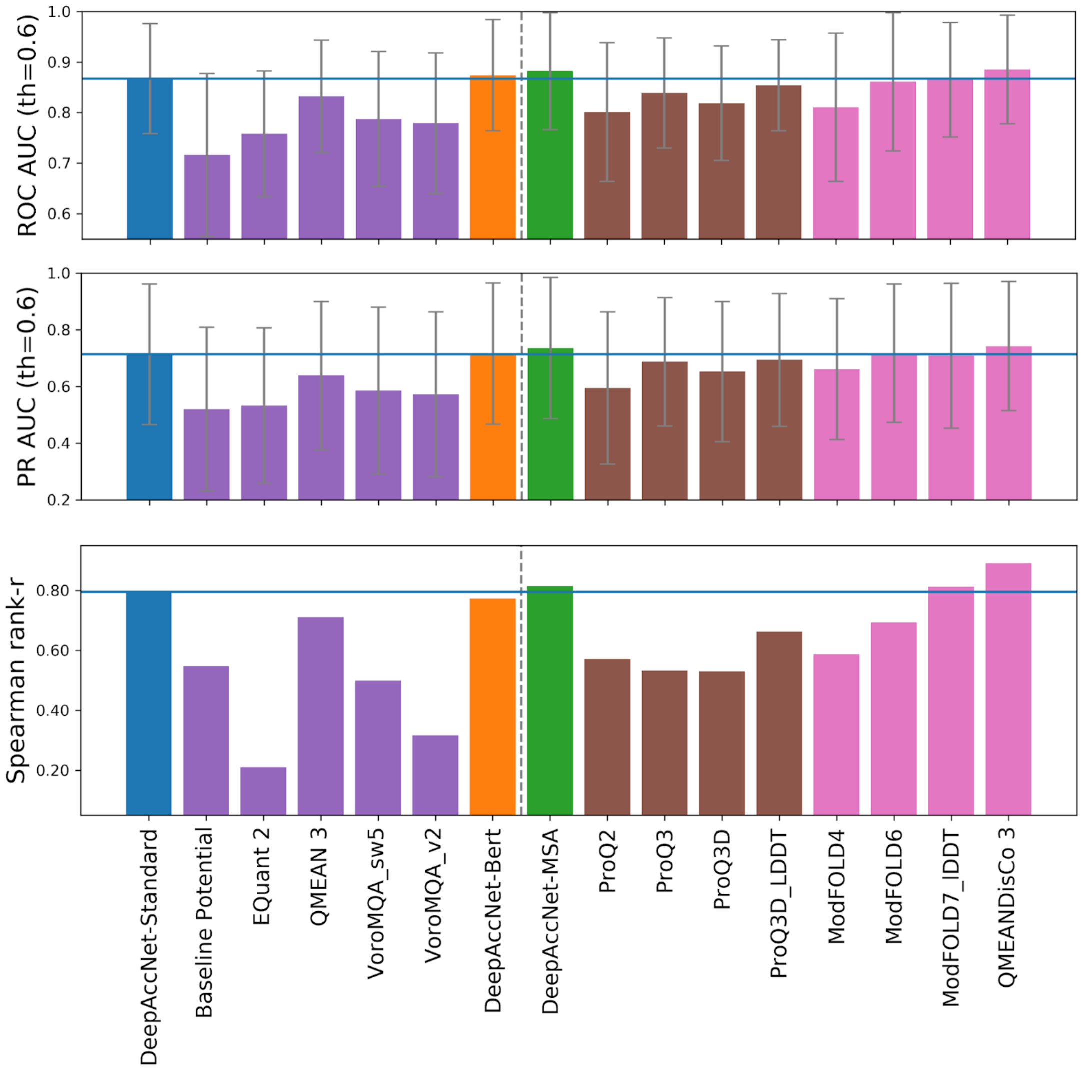

Supplementary Figure 5. Comparison of the performance of single model accuracy estimation (EMA) methods on CAMEO data. (Top, middle) Performance of local accuracy estimation measured by the mean of area under receiver operator characteristic (ROC, top) curve and precision-recall curve (PR, middle) for predicting mis-modeled residues per sample (all-atom l-DDT< 0.6). Error bars show standard deviation. (Bottom) Performance of global accuracy estimation measured by the mean of the Spearman correlation coefficient (*r*-value) of predicted and actual global l-DDT scores. Since the number of models per target was small, correlation was measured globally across all targets. The blue horizontal lines show the value of DeepAccNet-Standard. The methods to the left of the dotted line do not use coevolutionary information. Quasi-single models are shown in pink.

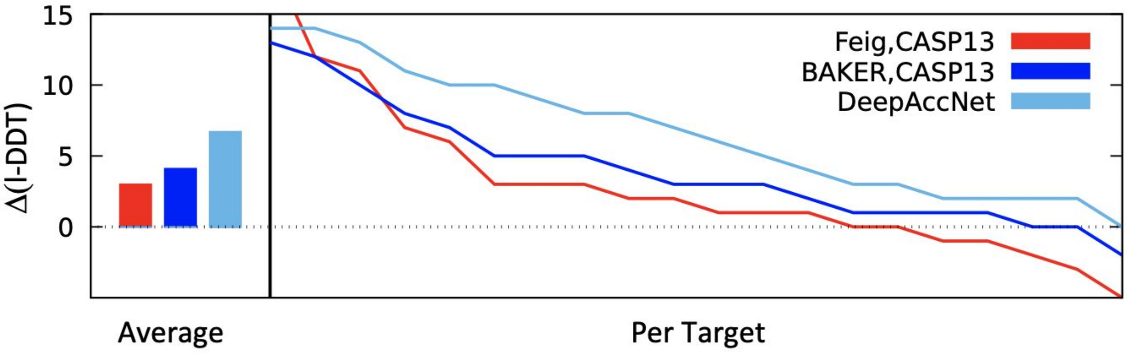

Supplementary Figure 6. Performances of the methods on CASP13 refinement category targets. Improvements in all-atom l-DDT scores over starting models are shown. Two leading groups in CASP13, Feig and Baker, are brought in for the comparison against refinement with DeepAccNet; Feig group ran long MD simulations, while BAKER group ran the non-DL refinement method presented in the main text with subsequent short MD simulations. Net all-atom l-DDT changes for both of these groups range within 3~4%, compared to 7% by DeepAccNet-guided refinement. 9 targets from the CASP13 refinement category are removed from the analysis for which the native structures contain heavy oligomeric contacts or are determined at low resolutions (>3 Å).

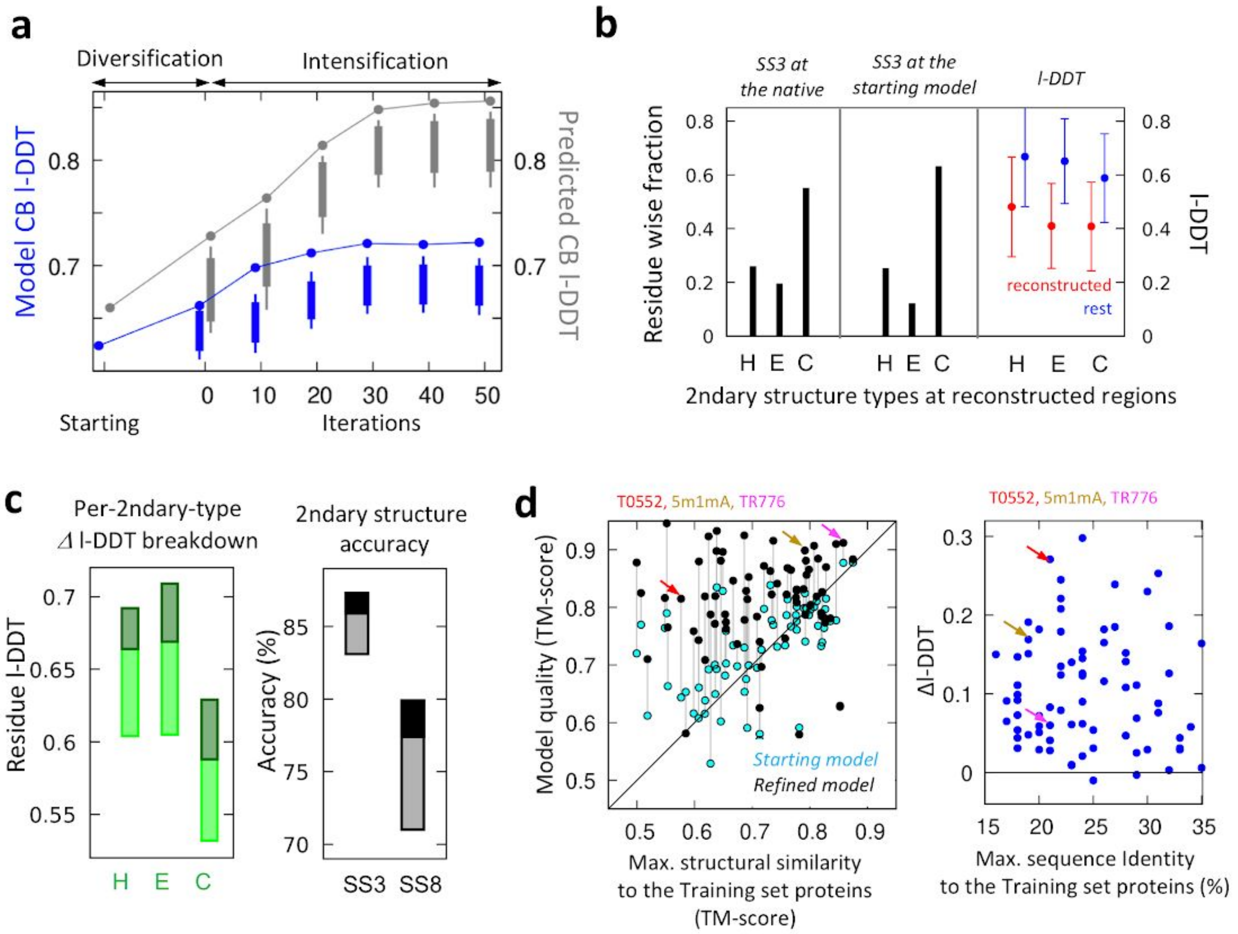

Supplementary Figure 7. Detailed analyses of refinement results. a) Actual and predicted model accuracy improvements throughout the refinement trajectory. Model quality (actual in blue and predicted in gray, C_β l-DDT is used for direct comparison), averaged over 73 benchmark cases, is shown through the refinement process. Points and bars show the model1 quality and the quality range of 50 models in the pool, respectively. **b**) 3-state secondary structure type at the reconstructed regions (H:helix, E:extended, C:coil). Residue-wise fractions of each type are plotted according to the native structure (left) and to the starting model structure (middle), respectively. (right) Pre-refinement l-DDT values at reconstructed regions and the rest preserved regions, shown in red and blue colors, respectively (average by circles; standard deviations by error bars). **c**) Breakdown of accuracy improvements by secondary structure types. Light colored boxes represent improvements without DeepAccNet-Standard, while darker regions of the boxes represent additional improvements gained with DeepAccNet-Standard; these are calculated over the complete benchmark set. (left panel) Similar improvements are observed across secondary structure types. (right panel) Improvements in model secondary structure accuracy are evaluated on 3- or 8-states following DSSP annotations [2]; improvements are evident in both 3 state and 8 state local structure prediction. (bottom panel) **d**) Correlation between refinement performance and highest structural/sequence similarity of the target to the training set proteins. (left panel). Correlation between the maximum structural similarity (x-axis) versus the starting/refined model quality (y-axis) shown in TM-score [3]. (right panel) Correlation between the maximum sequence identity (%) versus the refinement performance (in l-DDT change). In both panels, targets highlighted in Figure 4 are shown in colored arrows.

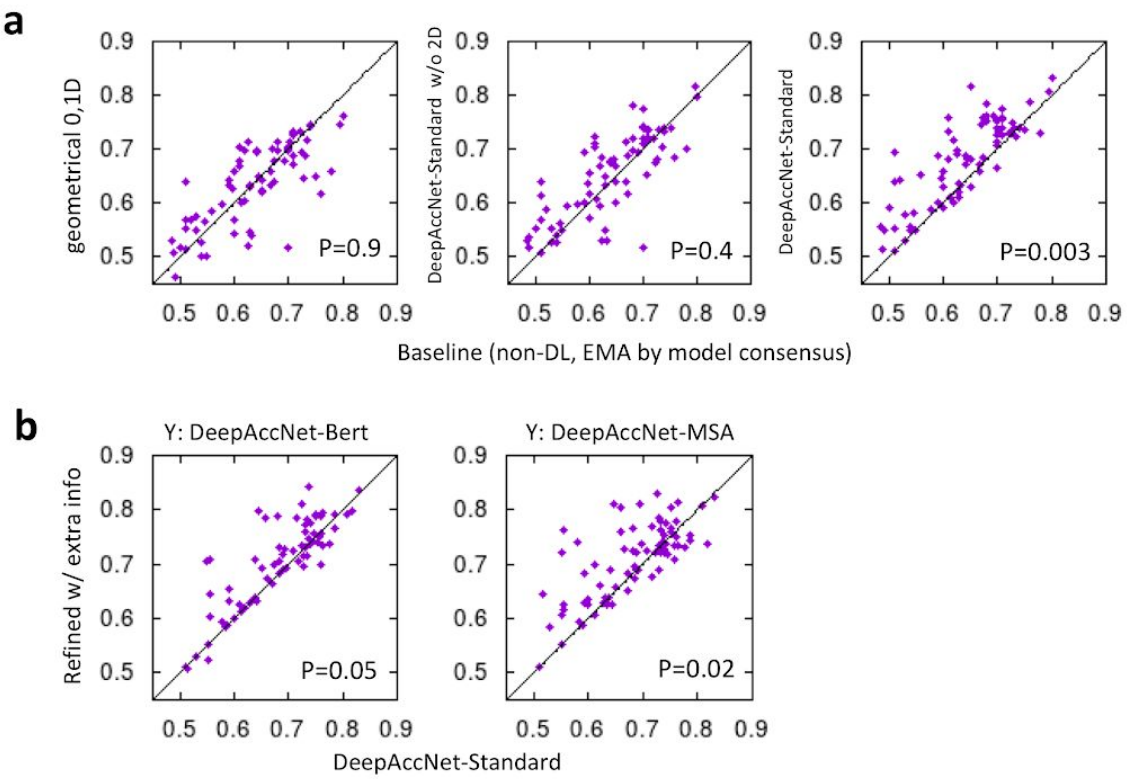

Supplementary Figure 8. Breakdown of Figure 4d: Comparison of refinement performances by EMA methods or extra information utilized. a) Refinement performance with different EMA methods taken during refinement, compared to that of our baseline approach (x-axis) [4,5] using model consensus for 1D (region detection) and 2D (residue pair confidence) and Rosetta energy for 0D (global ranking). **b**) Refinement performance gained by providing extra input from Bert and MSA features, compared to DeepAccNet without such extra input features (x-axis).

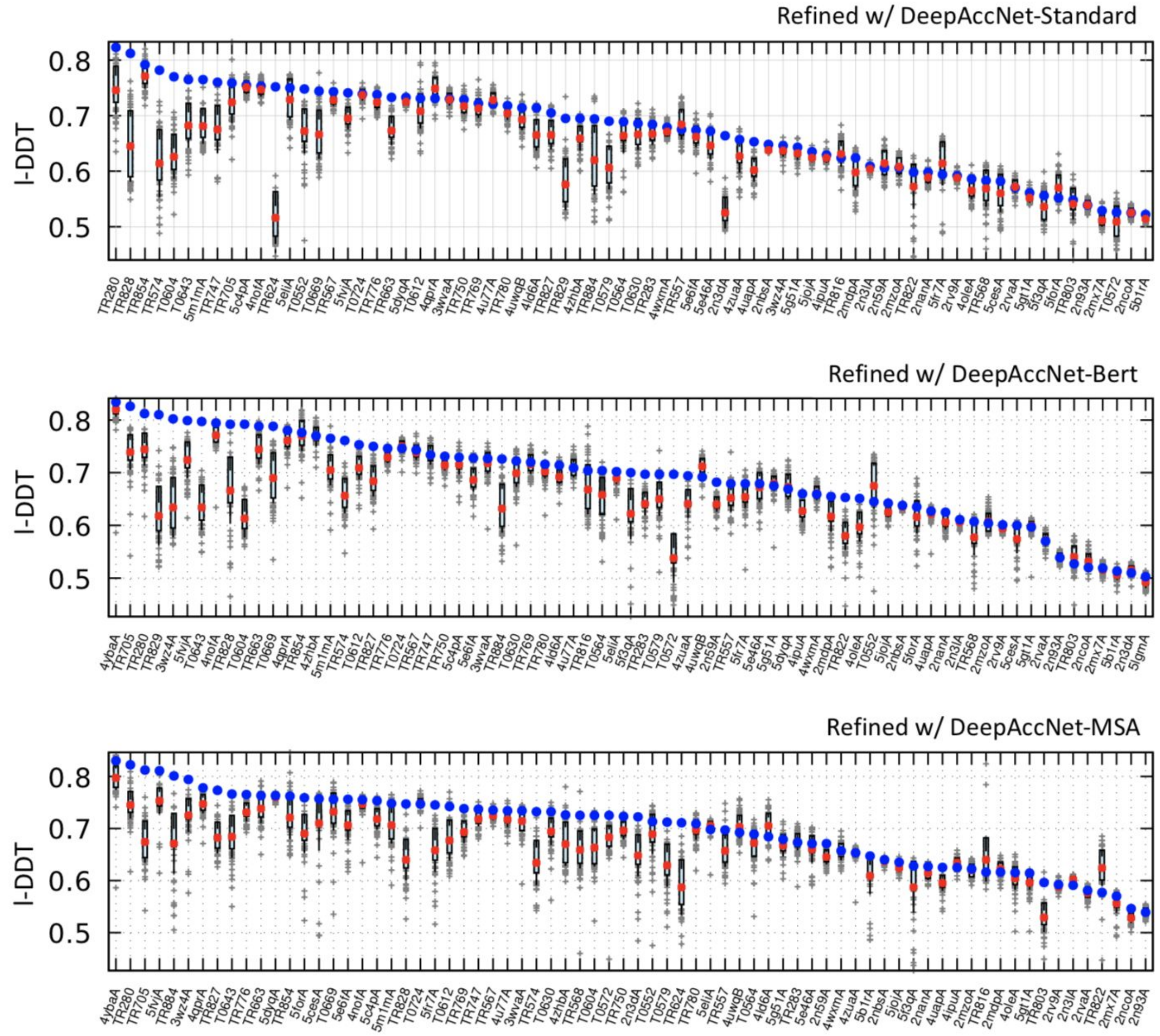

Supplementary Figure 9. The model quality of the final iteration structural pool and the selected one from the refinement runs using DeepAccNet-Standard, -Bert, and -MSA. 1st and 3rd quartile of the model qualities in the final iteration models shown in cyan bars, their mean in red dots, selected by DeepAccNet (without structural averaging) in blue dots, and individual values in gray crosses.

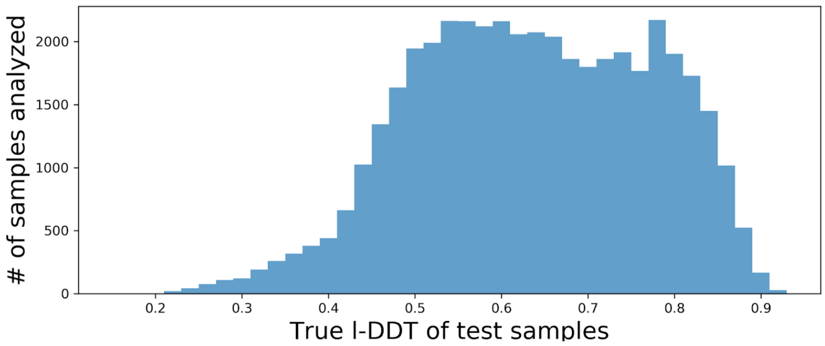

Supplementary Figure 10. Numbers of samples that participated in loss analysis based on starting l-DDT scores.

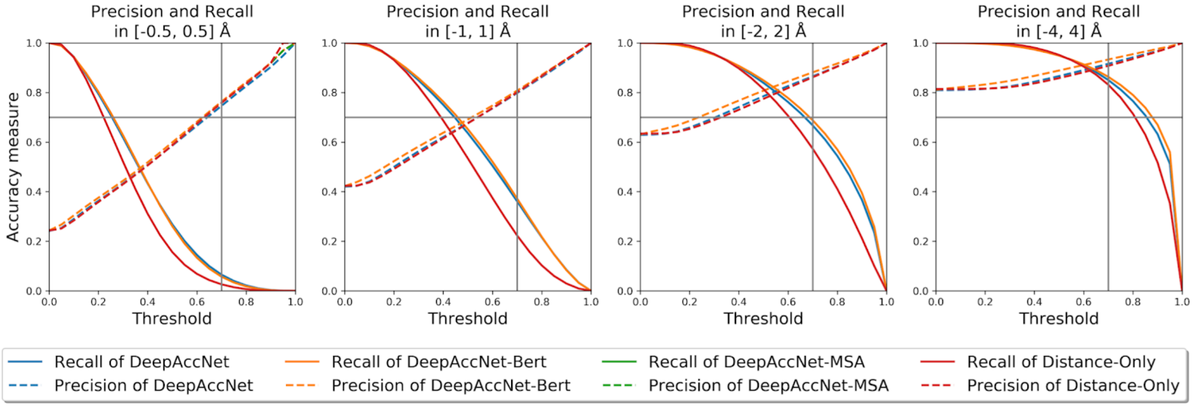

Supplementary Figure 11. Assessment of binary correct/incorrect predictions. Actual error values were grouped into correct and incorrect bins. In each panel, a distance is counted as correct if the actual distance error (from that of the native structure) is within a certain range, while a prediction is counted correct if the sum of probability over the given range in the estogram is above the threshold value (x-axis). Error range definitions are [-0.5, 0.5], [-1, 1] , [-2, 2], and [-4, 4] Å from the left to the right panel. The dotted lines show recall values and solid lines show precision values. The grey lines visualize the thresholding of 0.7 used in the downstream refinement process.

Supplementary Tables

Models	Held-out proteins (# proteins=285)			True global C_β l-DDT < 0.7			True global C_β l-DDT > 0.7		
	Esto	Mask	l-DDT	Esto	Mask	l-DDT	Esto	Mask	l-DDT
(i) DAN-Standard	1.805	0.200	0.012	1.939	0.250	0.014	1.567	0.110	0.009
(ii) DAN-Bert	1.697	0.171	0.009	1.781	0.208	0.010	1.548	0.106	0.009
(iii) DAN-MSA	1.557	0.135	0.008	1.594	0.158	0.009	1.489	0.094	0.008
(iv) C_β distance	1.901	0.217	0.017	2.022	0.270	0.017	1.685	0.123	0.016
(v) 3D conv	1.808	0.200	0.012	1.936	0.250	0.013	1.581	0.111	0.010
(vi) Bert	1.761	0.181	0.012	1.836	0.217	0.012	1.628	0.115	0.012
(vii) 3D+Bert	1.714	0.175	0.010	1.794	0.211	0.010	1.570	0.110	0.010
(viii) Rosetta	1.854	0.209	0.013	1.986	0.262	0.015	1.617	0.115	0.011
(ix) AA-related	1.863	0.208	0.014	1.977	0.258	0.014	1.659	0.119	0.014
(x) Sec struct	1.922	0.222	0.017	2.049	0.275	0.018	1.695	0.127	0.015
(xi) Angles and orientations	1.870	0.212	0.015	2.006	0.266	0.017	1.627	0.117	0.012

Supplementary Table 1: Performance of the variants of distance-based networks trained with and without a certain class of features. Performance is measured by cross-entropy for estograms and masks and mean squared error for C_β l-DDT scores. For each setting, we ensembled the prediction from four models with the best validation performance from the same training trajectory (see Methods). Columns 2-4 report the quality of the three predictions averaged over all held-out decoy structures. Columns 5-7 report the quality of the predictions on decoys with low true quality (global C_β l-DDT < 0.7). Columns 8-10 report the quality of the predictions on decoys with high true quality (global C_β l-DDT > 0.7). The decoys used for evaluation in columns 5-10 are subsets of the decoys used in columns 2-4.

**6B17, 3URO, 3TWG, 5DYR, 6HR0, 1P9G, 4G4L, 6EWN, 4HB6, 5JQF, 4U2W, 4HB8, 1MBN, 4HAJ, 1CYC, 1VXB, 3H4N, 2SBT, 1NXB, 4HBF, 1G7V, 2EWI, 1J0O, 2SNS, 4HDL, 3SJ4, 3H34, 4D5M, 1MBS, 1OS6, 2EWU, 1LWK, 1LYZ, 3TRV, 3SJ0, 4Z0W, 1ACX, 1PMK, 3TJW, 1HH5, 1M1R, 6DK5, 2ZVS, 3D6T, 2AOA, 3SEL, 6FM8, 5YP8, 4EFX, 1TGL, 3SJ1, 1TIA, 2EWK, 2XJI, 5HDD, 6CDX, 5VBD, 4HC3, 3NIR, 2YYX, 1HGU**

Supplementary Table 2: List of X-ray native structures with low C_β-lddt despite their high experimental resolution.

	Standard vs. Bert	Bert vs. MSA	Standard vs. MSA
**Test set (MSE loss of C_β l-DDT)** *1	p-value < 0.0001	p-value < 0.0001	p-value < 0.0001
**Test set (Cross-entropy loss of Estogram)** *2	p-value < 0.0001	p-value < 0.0001	p-value < 0.0001
**CASP13 (ROC AUC)** *3	p-value < 0.0001	p-value < 0.0001	p-value < 0.0001
**CASP13 (Spearman r)** *4	p-value < 0.0001	p-value < 0.0001	p-value < 0.0001
**CAMEO (ROC AUC)** *5	p-value < 0.0001	p-value < 0.0001	p-value < 0.0001
**CAMEO (PR AUC)** *6	p-value < 0.0001	p-value < 0.0001	p-value < 0.0001
**CAMEO (Spearman r)** *7	p-value = 0.069	p-value = 0.0003	p-value = 0.080

Supplementary Table 3: Significant tests to compare among the DeepAccNet variants. Wilcoxon signed-rank test was used to analyze *1~6 as the distribution of the difference between two variants's means is not assumed to be normally distributed. All differences in means are statistically significant between variants. For *7, we only have one r-value per variant unlike *6. Thus, we applied Fisher's Z transformation and analyzed the statistical significance based on the observed z test statistic.

Distance-based	i) C_β to C_β distance map, Cα is taken for GLY, ii) Cα to Tip-atom distance map and its transpose, iii) Tip-atom to Tip-atom distance map, and iv) sequence separation map. The distance maps (i~iv) go through a variance reduction process with *arcsinh(x).* See Supplementary Table 6 for the definition of tip atoms.
Amino acid properties	i) One-hot encoded amino acids. ii) Blosum62 scores [6]. iii) Per amino-acid feature sets from Meiler et al [7].
Rosetta energy terms	i) Two-body energy terms: fa_atr, fa_rep, fa_sol, lk_ball_wtd, fa_elec, hbond_bb_sc, and hbond_sc. ii) One-body energy terms: p_aa_pp, rama_prepro, omega, fa_dun. iii) Presence of backbone-to-backbone hydrogen bonds.
Backbone angles and lengths	i) Phi, Psi, and Omega angles. ii) Standardized length between backbone atoms.
residue-residue orientations	i) Full 6 degrees of freedom of translation and rotation. ii) cosine and sine of dihedral and planar angles defined by Yang et al [8].
Secondary structures	1-hot encoded representation of three state secondary structures given by DSSP solver.
Local atomic environments	24 by 24 by 24 voxels of size 0.8Å. In total, it covers an area of size 19.2Å by 19.2Å by 19.2Å. There are 20 channels for 20 atom types defined by Rosetta. The coordinate frame is fixed based on backbone N,Ca,C atoms [9].
Multiple sequence alignment	Inter-residue distance (30 by N by N, where N is protein size) predictions from trRosetta [8] gives indirect access to evolutionary multiple sequence alignments
Bert embeddings	Attention heads from the last attention layer of the ProtBert-BFD100 model [10] (16 by N by N, where N is protein size)

Supplementary Table 4: Generated features for all 9 major feature classes. Some features are scaled and normalized to a reasonable range. Please refer to the code available at github for further details on the normalization scheme.

Layers groups	Descriptions
3D convolution layers	This group has four layers of 3D convolution operations with 20, 20, 30, and 20 filters with sizes of 1, 3, 4, 4, respectively. Elu activation is used. Mean pooling of filter size 4 with stride 4 was performed at the end.
Feature merging	This operation merges flattened 3D conv outputs, 2D, and 1D features (**see Methods**). One layer of 2D convolution with 32 filters of size 1 and instance normalization are applied. Elu activation is then used. Finally, the output is upsampled to 256 channels for the following ResNet operations.
Residual blocks 1	Each residual block consists of (i) elu activation, (ii) projection down to 128 channels, (iii) elu activation layer (iv) 3 by 3 convolution, (V) elu activation, (vi) projection up to 256 channels. Instance normalization operations are applied. Residual connection adds inputs to (i) with outputs of (vi). 20 residual blocks are stacked. Dilation is applied to (iv) with a cycling dilation size of 1,2,4,8.
Residual blocks 2 for estograms and masks	Two arms of four residual blocks are applied to predict estograms and masks. The same numbers of channels (256-->128-->256) are used.
C_β l-DDT calculation layers	C_β l-DDT values are calculated within gpu memory based on predicted estograms and masks (**see Methods)**.
Loss	(i) Estograms are evaluated with categorical cross-entropy loss. (ii) Masks are evaluated with binary cross-entropy loss. (iii) l-DDT values are evaluated with mean squared loss. Global loss is defined and shown in Method.

Supplementary Table 5: Model architectures for the DeepAccNet. Please refer to the code available at github for further details on the implementation.

**amino acid**	**ALA**	**CYS**	**ASP**	**ASN**	**GLU**	**GLN**	**PHE**	**HIS**	**ILE**	**GLY**
**tip atom**	CB	SG	CG	CG	CD	CD	CZ	NE2	CD1	CA
**amino acid**	**LEU**	**MET**	**ARG**	**LYS**	**PRO**	**VAL**	**TYR**	**TRP**	**SER**	**THR**
**tip atom**	CG	SD	CZ	NZ	CG	CB	OH	CH2	OG	OG1

Supplementary Table 6: Definitions of tip atoms for each residue.

Supplementary References

1. **Mirdita, M. *et al.* Uniclust databases of clustered and deeply annotated protein sequences and alignments. *Nucleic Acids Res.* 45, D170–D176 (2017).**
2. **Kabsch, W. & Sander, C. Dictionary of protein secondary structure: pattern recognition of hydrogen-bonded and geometrical features. *Biopolymers* 22, 2577–2637 (1983).**
3. **Zhang, Y. & Skolnick, J. TM-align: a protein structure alignment algorithm based on the TM-score. *Nucleic Acids Res.* 33, 2302–2309 (2005).**
4. **Park, H. *et al.* High-accuracy refinement using Rosetta in CASP13. *Proteins: Structure, Function, and Bioinformatics* vol. 87 1276–1282 (2019).**
5. **Park, H., Ovchinnikov, S., Kim, D. E., DiMaio, F. & Baker, D. Protein homology model refinement by large-scale energy optimization. *Proc. Natl. Acad. Sci. U. S. A.* 115, 3054–3059 (2018).**
6. **Henikoff, S. & Henikoff, J. G. Amino acid substitution matrices from protein blocks. *Proc. Natl. Acad. Sci. U. S. A.* 89, 10915–10919 (1992).**
7. **Meiler, J., Zeidler, A., Schmaschke, F. & Muller, M. Generation and evaluation of dimension-reduced amino acid parameter representations by artificial neural networks. *Journal of Molecular Modeling* vol. 7 360–369 (2001).**
8. **Yang, J. *et al.* Improved protein structure prediction using predicted interresidue orientations. *Proc. Natl. Acad. Sci. U. S. A.* 117, 1496–1503 (2020).**
9. **Pagès, G., Charmettant, B. & Grudinin, S. Protein model quality assessment using 3D oriented convolutional neural networks. *Bioinformatics* 35, 3313–3319 (2019).**
10. **Elnaggar, A. *et al.* ProtTrans: Towards Cracking the Language of Life's Code Through Self-Supervised Deep Learning and High Performance Computing. doi:10.1101/2020.07.12.199554.**
